# Reply to Urbina, F.; Benavides, A. Telangiectatic Mastocytosis: If It Is Not Mastocytosis, What Is It? Comment on “Brockow et al. Challenges in the Diagnosis of Cutaneous Mastocytosis. *Diagnostics* 2024, *14*, 161”

**DOI:** 10.3390/diagnostics15111371

**Published:** 2025-05-29

**Authors:** Knut Brockow, Rebekka Karolin Bent, Simon Schneider, Sophie Spies, Katja Kranen, Benedikt Hindelang, Zsuzsanna Kurgyis, Sigurd Broesby-Olsen, Tilo Biedermann, Clive E. Grattan

**Affiliations:** 1 Department of Dermatology and Allergy Biederstein, School of Medicine and Health, Faculty of Medicine, Technical University of Munich, Biedersteiner Strasse 29, 80802 Munich, Germanysimon.schneider@mri.tum.de (S.S.); sophie.spies@mri.tum.de (S.S.); katja.kranen@mri.tum.de (K.K.); benedikt.hindelang@tum.de (B.H.); zsuzsanna.kurgyis@tum.de (Z.K.); tilo.biedermann@tum.de (T.B.); 2 Department of Dermatology and Allergy Centre, Odense University Hospital, 5000 Odense, Denmark; sigurd.broesby-olsen@rsyd.dk; 3 St John’s Institute of Dermatology, Guy’s Hospital, London SE1 9RT, UK; clive.e.grattan@gstt.nhs.uk

We appreciate the interest and reflections of Urbina and Benavides on our recent article published in *Diagnostics* and presentation of their two new patients [1]. They are correct in stating that the histological criteria of mastocytosis have not been well defined and that mast cell (MC) counts per mm^2^ or per high power field (HPF) do not always discriminate between patients with mastocytosis as compared to patients with other dermatological diseases, pruritus or healthy controls [2,3]. However, we oppose their proposal that not meeting histological criteria but at the same time not fulfilling defined exclusion criteria should be sufficient for giving a definitive diagnosis. This may lead to confusion concerning disease definitions and may impede progress in the recognition of not yet described diseases or symptom constellations. Although large textbooks and the literature may cover the majority of disease manifestations, some patients do not fit well to any of these diagnoses. Either they stand in between two diseases (e.g., “eczematous psoriasis”) or show a quite individual manifestation and symptom pattern, which requires individual treatment deduced from the therapy recommendations of the most similar disease.

The two patients presented by Urbina and Benavides were both described as having urticarial dermographism. This is an exaggerated wheal and flare response that occurs within minutes of the skin being stroked or scratched on any part of the skin and is different from Darier’s sign in that the latter is restricted to the skin lesions of mastocytosis, but not to uninvolved skin. Symptomatic dermographism is the most common form of chronic inducible urticaria, which is caused by activated mast cells [4]. In the first patient with an unspecified bleeding disorder and epistaxis, dermographism may have led to pruritus, subconscious scratching and ecchymosis. The second patient had additional pruritus and diarrhoea with episodes of tachycardia as well as telangiectasias: systemic mast cell activation should be looked for. Hamilton et al. reported a few patients who presented not only with symptoms and a response to anti-mast cell mediator therapy such as antihistamines, but also with an increase in mast cell mediators, e.g., tryptase, during an episode [5]. Abdominal pain (94%), dermographism (89%), flushing (89%) together with headache (83%) were possible indicators, of which the second patient described had at least two of these symptoms. Neither patient met typical clinical findings of mastocytosis, which are maculopapular skin lesions, increased basal serum tryptase levels, activating D816V *KIT* mutation, insect venom anaphylaxis or unexplained osteoporosis. As the presented histologies, together with an ambivalent symptomatology, are insufficient to confirm mastocytosis, the activating D816V *KIT* mutation should be looked for and demonstrated in the skin biopsy specimen, bone marrow or peripheral blood, before such a diagnosis is made [6].

There is an association between MC and blood vessels and vice versa [7]. At the NIH, one of the authors (KB) noted that rosacea-like skin lesions and telangiectasias in addition to more typical lesions of maculopapular cutaneous mastocytosis (MPCM) were quite common in patients with mastocytosis. This may be due to the MC production of angiogenic factors such as vascular endothelial growth factor [7]. However, this association also exists the other way round, as in a histology of healthy skin, MCs mostly cluster around vessels, glands and nerves, which appear to associate with MCs [3]. Thus, it is not surprising that essential telangiectasias are surrounded by increased numbers of mast cells and, thus, found on a histology. In a study on 24 patients admitted to the NIH with the diagnosis of telangiectasia macularis eruptiva perstans (TMEP) by referral and/or by histology, most patients lacked overt telangiectasias, and their final diagnosis was highly vascularized urticaria pigmentosa/MPCM [8]. All patients had cutaneous symptoms (urticaria, pruritus, or flushing), and 75% of them additional extracutaneous symptoms. Tryptase levels were elevated in all patients (median 24.2 ng/mL; interquartile range, 14.7–48.6 ng/mL), which differed from the two patients described by Urbina and Benavides. This is in agreement with the original description of TEMP by Weber and Hellenschmied, who noted erythematous nonpurpuric macules, which disappeared under diascopic pressure, but without visible telangiectasia (‘broken veins’) by naked eye examination. They could not find an explanation other than “telangiectatic hyperemia” [9]. This may have been misinterpreted to indicate that individual telangiectasia can be seen with the naked eye as evidence of TMEP, which is not the case. It was the collective opinion of the European Competence Network on Mastocytosis (ECNM) that the term and definition of TMEP as a subvariant of cutaneous mastocytosis is no longer supported and that telangiectasia alone without other more typical MPCM lesions are not sufficient for the diagnosis of mastocytosis. Thus, the subvariant of TMEP was removed from the classification [10,11].

Several valid questions were raised by Urbina and Benavides. Although MC numbers differ with body location, we do not believe that biopsies from other sites or further biopsies would have helped to make the diagnosis. Furthermore, some details may lead to strong methodological differences. In the publication by Gebhardt et al., only MCs were counted in which the nucleus was visible (“nucleated MC”), whereas this was not mentioned in the comment we are reviewing and might have led to much higher MC numbers [3]. Numbers in the publications always refer to specific patient populations studied and should be used as a general guideline; however, exceptions may exist, for example, when studying tissue with a lot of vessels or glands. A histology has to be interpreted alongside clinical features. Increased perivascular or periadnexal numbers alone have to be regarded with caution as they are not diagnostic by themselves. Finally, regarding the questions on recurrent or persisting symptoms in different organ systems as possible signs for mastocytosis, it is true that such symptoms may accompany systemic mastocytosis. However, without additional specific typical features of mastocytosis (as indicated at the end of the first paragraph above), such symptoms are actually much more common in patients who visit our outpatient clinics and in whom we cannot confirm this diagnosis, and thus, these symptoms are rather unspecific. A positive bone marrow biopsy can prove or exclude systemic mastocytosis. However, we would consider this diagnosis in the two patients presented unlikely and would rather recommend determining the D816V *KIT* variant less invasively in peripheral blood or in skin biopsy specimen as a screening test for exclusion, if available.
